# Morphological and molecular characterization of *Cyclopodia* bat flies (Diptera: Nycteribiidae) from Australian *Pteropus* hosts

**DOI:** 10.1017/S0031182026101929

**Published:** 2026-04

**Authors:** Robyn T. Pearce, Constantin Constantinoiu, Kaitlin V. Janssen-Groesbeek, Varsha V. Balu, Roslyn I. Hickson, Anjana C. Karawita, Paul F. Horwood

**Affiliations:** 1 Australian Institute of Tropical Health and Medicine, James Cook University, Townsville and Cairns, QLD, Australia; 2 College of Science and Engineering, James Cook University, Townsville and Cairns, QLD, Australia; 3 Commonwealth Scientific Industrial Research Organisation (CSIRO), Townsville, QLD, Australia; 4 Commonwealth Scientific Industrial Research Organisation (CSIRO), Geelong, VIC, Australia; 5 Centre for Tropical Biosecurity, James Cook University, Townsville and Cairns, QLD, Australia; 6 College of Medicine and Dentistry, James Cook University, Townsville and Cairns, QLD, Australia

**Keywords:** *Cyclopodia albertisii*, *Cyclopodia australis*, ectoparasites, host specificity, Nycteribiidae, *Pteropus*

## Abstract

Bat flies (Diptera: Nycteribiidae and Streblidae) are obligate blood-feeding ectoparasites of bats and are increasingly recognised for their potential role in host-specific co-evolution and disease transmission. Despite their ecological importance, the diversity, host associations and evolutionary relationships of bat flies in Australia remain poorly characterised. This study provides the first integrative assessment of nycteribiid bat flies parasitising *Pteropus* species in North Queensland, combining morphological taxonomy with cytochrome c oxidase subunit 1 (COX1) sequencing and phylogenetic analysis. A total of 304 bat flies were collected from 79 rescued pteropodids, representing three host species: the little red flying fox (*Pteropus scapulatus)*, the black flying fox *(Pteropus alecto)* and the spectacled flying fox *(Pteropus conspicillatus)*. Morphological examination of the bat flies identified two taxa, *Cyclopodia australis* and *Cyclopodia albertisii*, which were further confirmed by maximum likelihood phylogenetic analysis of COX1 sequences with distinct clade formations delineating species. *C. australis* was found almost exclusively on little red flying foxe*s. C. albertisii* was mainly associated with both black flying foxes and spectacled flying foxes, with very limited association with the little red flying foxes. These findings underscore the utility of integrative taxonomic approaches in researching bat fly diversity and host specificity. They also highlight the potential for co-evolutionary divergence and emphasise the need for expanded geographic sampling and genomic analysis. This research provides critical baseline data for understanding ectoparasite biodiversity in Australia and contributes to future studies of host–parasite interaction, vector ecology and wildlife disease surveillance.

## Graphical abstract

**Figure:**
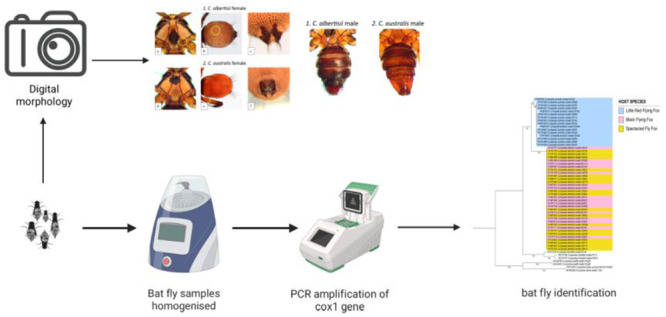

## Introduction

Bats (Order: Chiroptera) are among the most ecologically diverse and geographically widespread mammalian groups, with 1500 recognized species worldwide (Simmons and Cirranello, 2025). They are classified into 2 suborders, Yangochiroptera and Yinpterochiroptera, the latter of which contains all fruit bats and flying foxes (family Pteropodidae) plus 5 additional bat families (Teeling et al., 2005). In Australia, pteropodids play vital roles in ecosystem health by acting as pollinators and seed dispersers (Irving et al., 2021), particularly in tropical and subtropical regions. In North Queensland, prominent pteropid species include the black flying fox (*Pteropus alecto*), the little red flying fox (*Pteropus scapulatus*), and the spectacled flying fox (*Pteropus conspicillatus*) (Churchill, 2008). The grey-headed flying fox (*Pteropus poliocephalus*) is more common in southern Australia, but can migrate north during winter seasons (Churchill, 2008). These 4 species are highly social, forming large colonies that enable complex social behaviour, offer protection, and facilitate mating (Pierson and Rainey, 1992; Lunn et al., 2021). This communal behaviour also promotes the transmission of pathogens, including zoonotic viruses (Hengjan et al., 2017; Hemamalani et al., 2025), and ectoparasites like bat flies (Szentiványi et al., 2019). Given their ecological mobility (Welbergen et al., 2020), unique immune tolerance to viruses, and frequent contact with both wildlife and human environments, bats are increasingly recognised as key reservoirs of emerging infectious diseases (Calisher et al., 2006). Their obligate ectoparasites, such as bat flies, may act as secondary carriers or bridging vectors for a wide range of pathogens. Through their strong host specificity, bat flies could contribute to species-specific pathogen maintenance and transmission (Dick and Patterson, 2006). Rare human and animal bites have been reported, and bat flies could represent a potential interface for spillover between bats, other animals, and humans (Dick and Patterson, 2006; Szentiványi et al., 2019; Szentivanyi et al., 2023).

Bat flies (Diptera: Hippoboscoidea) are obligate blood-feeding ectoparasites belonging to the families Nycteribiidae and Streblidae (Dick and Patterson, 2006). They exhibit high host specificity and a close relationship with their bat hosts (Dittmar et al., 2006; Patterson et al., 2007). Bat flies are highly adapted to a parasitic lifestyle and possess specialised morphological features such as flattened bodies, reduced or absent wings, and strong claws for clinging to bat fur (Lee et al., 2021). Their life cycle is tightly linked to the host, with individuals spending most of their lives on the bat’s body. Male bat flies rarely leave the host, while females only leave to give birth to a single larva, which immediately pupates in the roost environment. As soon as the adult fly emerges from the puparium, it finds a bat host and immediately begins blood-feeding (Dittmar et al., 2006). Due to their intimate, long-term relationship with bat hosts and their dependence on blood meals, bat flies serve as potential reservoirs and vectors for various pathogens, including bacterial species such as *Bartonella* and *Rickettsia*, as well as viral pathogens such as dengue virus (DENV) (Morse et al., 2012; Wilkinson et al., 2016; Abundes-Gallegos et al., 2018; Xu et al., 2022; Szentivanyi et al., 2023). This positions them as indirect indicators of pathogens present within a bat colony and as potential vectors of zoonotic concern.

There is strong evidence of host specificity (Ramirez-Martinez et al., 2021; Poon et al., 2023; ) among bat fly species, and accurate identification is essential to understand their ecological roles and potential as vectors in disease transmission. Accurate species identification is also fundamental for assessing their diversity and evolutionary relationships with their hosts. Morphological identification, however, is limited by intraspecific variation, sexual dimorphism and similarity among closely related species (Dick and Patterson, 2006). Some morphological traits used for species identification, including body size, wing venation (the pattern of veins on the wing) and genital anatomy, can vary significantly between sexes and within populations of the same species (Dick and Patterson, 2006; Lee et al., 2021). This variation can lead to misidentification and underestimation of species diversity. Furthermore, the small size and delicate nature of bat flies present additional challenges for accurate morphological classification under traditional examination (Lee et al., 2021). To overcome these limitations, molecular tools have become increasingly important for confirming species identity and detecting hidden diversity in bat flies. While morphological analysis provides valuable insights into external characteristics and evolutionary patterns, molecular data offer a robust and complementary method for confirming species identity and detecting diversity (Dittmar et al., 2009). The cytochrome c oxidase I (COX1) gene is widely used for bio-identification of insect species (Hebert et al., 2003) and has proven effective in identifying bat flies and other blood-feeding ectoparasites, distinguishing closely related taxa, and revealing cryptic species (Attaullah et al., 2023; Najera-Cortazar et al., 2023). An integrated approach that combines morphological and molecular data improves taxonomic resolution, reduces misidentification and enhances our understanding of bat fly diversity, host specificity and ecological function (Dittmar et al., 2009).

In Australia, the morphology and diversity of bat flies associated with Australian pteropodids remain poorly characterised. Previous morphological studies, with limited numbers of specimens, have been conducted in South America (Braga et al., 2020; Alcantara et al., 2022; Speer et al., 2022; Andre et al., 2023; Mejia et al., 2023), Africa (Ramirez-Martinez et al., 2021; Kamani et al., 2022; Atobatele et al., 2023; Bendjeddou et al., 2023; Szentivanyi et al., 2023) and Asia (Abundes-Gallegos et al., 2018; Ramirez-Martinez et al., 2021; Low et al., 2022; Fagre et al., 2023; Poon et al., 2023), with limited sampling and molecular characterisation of Australian taxa (Archer and Cardinal, 2001; Vidgen et al., 2017; Holz et al., 2018; Kwak et al., 2022). These studies suggest that further investigations using larger specimen collections are needed to resolve fine morphological features. Bat flies from different geographical regions may have evolved distinct genetic lineages through processes such as geographic isolation, host specialisation, and co-adaptation. Phylogenetic studies are therefore critical to clarify the evolutionary relationships between Australian bat flies and their global counterparts. Resolving these relationships will enhance our understanding of the biogeography of bat fly evolution and the extent of host–parasite co-evolution in Australia (Holz et al., 2018).

The objectives of this study were to: (1) identify and characterise bat fly species collected from 3 *Pteropus* host species; (2) assess host–parasite associations; and (3) infer phylogenetic relationships of isolated bat flies based on COX1 gene sequences.

## Materials and methods

### Specimen collection

A total of 304 bat flies were collected from 79 pteropodids spanning 3 species: the black flying fox (*P. alecto*), spectacled flying fox (*P. conspicillatus*) and the little red flying fox (*P. scapulatus*), rescued from the Townsville and Atherton Tablelands regions of North Queensland, Australia, between December 2023 and March 2025. The study area encompasses tropical and subtropical environments along Australia’s northeast coast, with Townsville located in the coastal lowlands and the Atherton Tablelands representing elevated tropical rainforest habitat approximately 100 kilometres inland. Bat and bat flies were collected throughout an approximately 400 km diameter area. Bat fly collection was conducted by trained wildlife carers from Townsville Bat Rescue (3 carers), NQ Wildlife Rescue and Rehabilitation (Townsville; 1 carer) and the Tolga Bat Hospital. Carers removed the flies from individual bats using forceps. Information on bat sex, age, body condition, collection site and time of collection was recorded by the trained carers using their established criteria. These data are available on request. Parasite sampling was not exhaustive, with collection efforts often limited to 5 specimens per host individual, regardless of total parasite burden. Each fly was preserved in separate microtubes containing DNA/RNA Shield (Zymo Research Corporation, California, USA) and stored at 4°C prior to laboratory processing at the Australian Institute of Tropical Health and Medicine (AITHM), James Cook University, Townsville, QLD.

### Morphological identification

**Figure 1.**
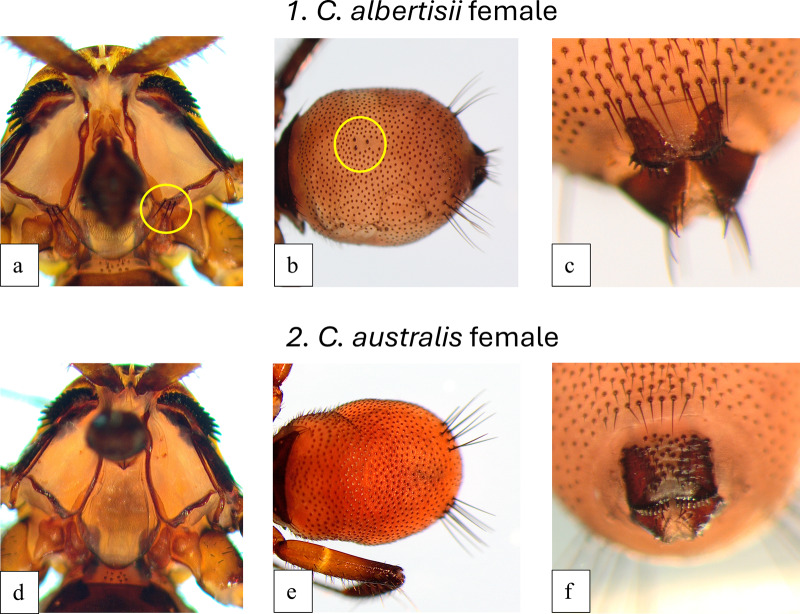
Female bat fly morphological features used for species identification and differentiation. (1) *C. albertisii*. (a) Thorax with notopleural setae. (b) Abdomen, dorsal view showing thicker blunt spines in centre of bare area and posterior setae formation. (c) Genital plate. (2) *C. australis*. (d) Thorax without notopleural setae, (e) Abdomen, dorsal view with no bare area and posterior setae formation. (f) Genital plate. Figure 1 long description.

**Figure 2.**
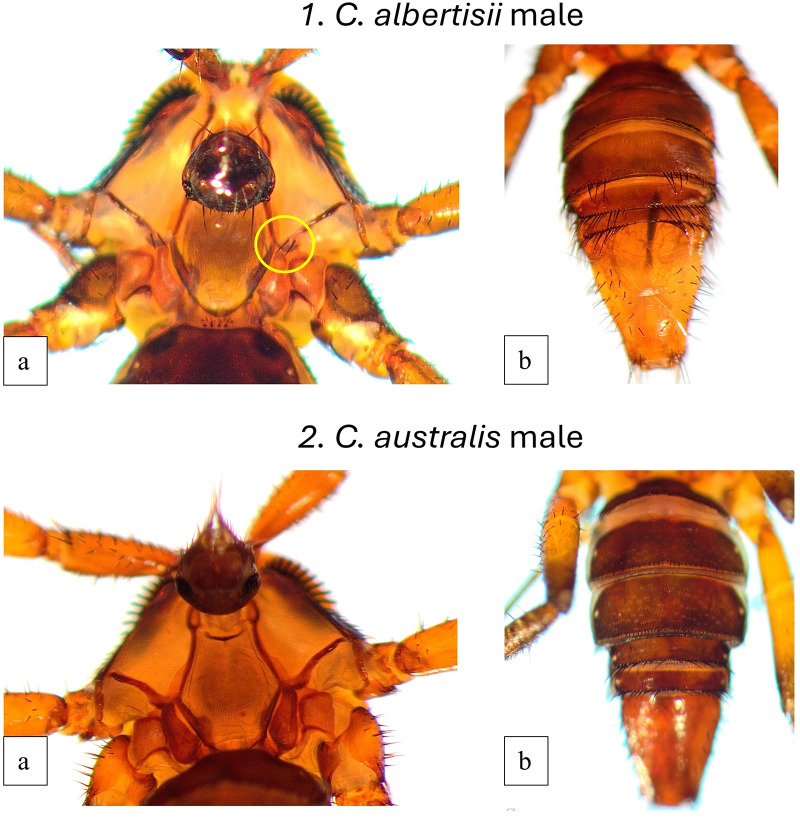
Male bat fly morphological features used for species identification and differentiation. (1) *C. albertisii*. (a) Thorax with notopleural setae. (b) Abdomen, dorsal view showing setae present on the lateral region of tergite 3, 4, 5 and 6. (2) *C. australis*. (c) Thorax without notopleural setae. (d) Abdomen, dorsal view showing setae absent on tergite 3, present only on the middle region of tergite 4, and across the entire posterior edge of tergite 5 and 6. Figure 2 long description.

Bat flies were washed in 80% ethanol and examined using a dissecting stereomicroscope (SZX10, Olympus Corporation, Tokyo, Japan) fitted with a microscope camera (SC50, Olympus Corporation, Tokyo, Japan). Morphological characteristics for species identification were determined using modified published taxonomic keys (Theodor, 1959; Theodor and Rothschild, 1967; Maa, 1971; Allison, 1987; Graciolli and Dick, 2018) to accommodate distinct physical differences between males and females and to improve field identification efficiency (see Appendices I and II). The flies were divided based on their sex and morphotype. A subset of bat flies were photographed. Morphological identification of each bat fly species included the presence or absence of notopleural setae (both female and male specimens; Figure 1a, d; 2a, b). For female specimens, the presence/absence of a bare patch on the dorsal abdomen and posterior setae formation (Figure 1b, e), the shape of the genital plate (Figure 1c, f), and the overall pigmentation were used for species separation. For males, the sclerotised plates and setae arrangements (Figure 2) were used as identifying features.

### COX1 amplification

Using a random number generator (Graphpad QuickCalcs Website: http://www.graphpad.com/quickcalcs/ConfInterval1.cfm accessed March 2025) 10% of males and 10% of females were selected from each distinct species determined morphologically. Any samples that exhibited variation in morphological features were also selected for COX1 amplification. Genomic DNA was extracted using the DNeasy Blood and Tissue Kit (Qiagen, USA) following the manufacturer’s protocol for insect extraction with modifications. Briefly, individual flies were washed in 80% ethanol and homogenised using disposable microtube pestles (NEB #T3002) in PBS with 20 µL of proteinase K, then incubated at 56°C for 10 minutes. To enhance DNA yield, the samples were eluted with 100 µL AE elution buffer and passed through the same column twice. Concentration was assessed via NanoDrop 2000c spectrophotometer (Thermo Fisher Scientific Inc., V1.6.198).

A 636-bp fragment of the COX1 gene was amplified using Qiagen HotStarTaq Plus Master Mix (Qiagen, Hilden, Germany, cat. No. 203645) and primers LC01490-F (5-GGTCAACAAATCATAAAGATATTGG-3′) and HC02198-R (5′-TAAACTTCAGGGTGACCAAAAAATCA-3′) (Ramirez-Martinez et al., 2021; Poon et al., 2023). The reaction volume included 25 µl HotStarTaq Plus Master Mix 2X, forward and reverse primers (10 µM each), 2 µl template DNA and nuclease-free water to make a final reaction volume of 50 µl (Attaullah et al., 2023). The Applied Biosystems SimpliAmp Thermal Cycler was used following the optimised conditions adapted from Ramirez-Martinez et al. (2021). PCR conditions included an initial heat activation of HotStarTaq Polymerase of 5 min at 95°C, followed by 35 cycles of 94°C for 30 seconds, 50°C for 30 seconds, 72°C for 1 minute, with a final extension at 74°C for 10 minutes. Negative controls were included to assess for contamination. PCR products were visualised on a 1.5% agarose gel using the InvitrogenTM 1Kb Plus DNA Ladder (Thermo Fisher Scientific Inc., Cat. No. 10787018) and sequenced in both directions by capillary electrophoresis at Macrogen, Inc. (Seoul, South Korea).

### Sequencing and phylogenetic analysis

**Figure 3.**
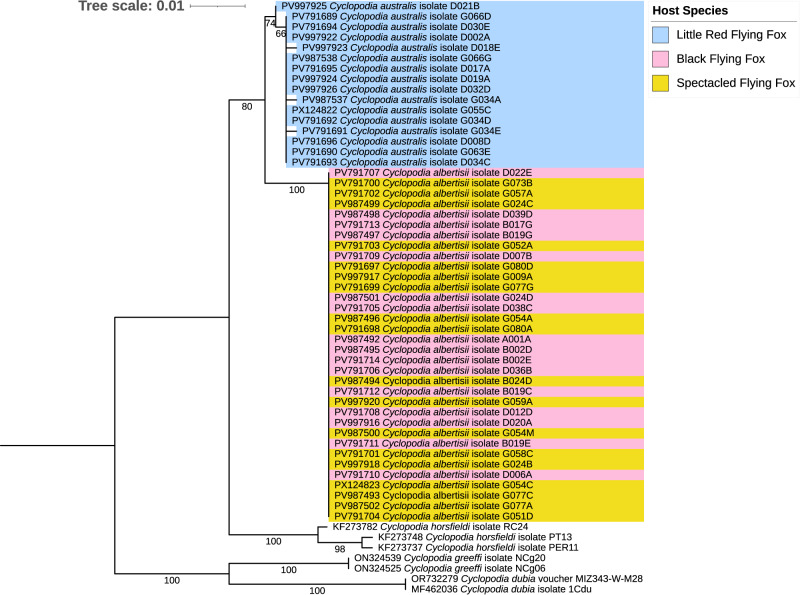
Evolutionary analysis by the maximum likelihood method. Maximum likelihood phylogenetic tree of Cyclopodia bat flies constructed using COX1 mitochondrial gene sequences. Sequences were obtained from individuals collected from three Pteropus host species in North Queensland: The little red flying fox (blue), spectacled flying fox (yellow) and black flying fox (pink). Two well-supported clades were recovered corresponding to C. australis and C. albertisii, confirming species-level delineation. Outgroup sequences from *C. horsfieldi, C. dubia* and *C. greeffi* are included to root the tree. Tree scale represents substitutions per site. Figure 3 long description.

Forward and reverse COX1 sequences from 56 samples were analysed using Geneious Prime (v 2025.1.3, GraphPad Software LLC). From 112 reads, 8 reads from 4 samples could not be assembled, with 2 additional sequences excluded due to low read quality. The remaining 50 high-quality sequences were trimmed, *de novo* assembled and aligned using the Geneious assembler in Geneious Prime v2025.1.3. Seven additional sequences downloaded from the National Center for Biotechnology Information GenBank database were included to serve as reference groups based on known geographic homology. *C. horsfieldi* (Accession numbers: KF273782, KF273748 and KF273737) is found in the Asia Pacific Region, *C. greeffi* (ON324539 and ON324525) is found on mainland Africa and Gulf of Guinea islands (Theodor and Rothschild, 1967) and *C. dubia* (MF462036 and OR732279) is found in Madagascar. The sequences generated from this study were deposited in GenBank (Accession numbers in Fig. 3). A Maximum Likelihood (ML) phylogenetic tree was constructed in MEGA v12.0.9 using the Tamura-Nei model with invariant sites (TN93 + I). This model was selected using Bayesian Information Criterion (BIC) scores. Bootstrap analysis (1000 replicates) supported the branch assessment. The tree was visualised and edited with iTOL v7.2. Bat fly species with distinct sequences were cross-checked with the morphological descriptions and published illustrations presented in the literature (Theodor and Rothschild, 1967; Poon et al., 2023).

## Results

### COX1 sequencing and characteristics

From 56 specimens selected for molecular analysis, including samples showing differences in reported morphological characteristics, 50 high-quality COX1 sequences suitable for phylogenetic reconstruction (89.3% success rate) were analysed. All sequences exhibited a G-C content of 24.8–26.3%, which is consistent with typical insect mitochondrial genome composition (Sweet et al., 2021; Grant et al., 2024). Sequence lengths ranged from 658 to 691 base pairs after quality trimming and alignment. Across the range of 658–691 base pairs analysed *C. albertisii* individuals displayed 100% sequence identity while *C. australis* ranged from 99.64% to 100% sequence identity. The sequence identity between *C. albertisii* and *C. australis* was 98.34% to 98.72%.

### Species identification and morphological characterization

Of the 304 bat fly specimens, 168 were identified to be male and 136 female. Morphological examination revealed 2 distinct taxa, *C. albertisii* and *C. australis*, within the family Nycteribiidae, subfamily Cyclopodiinae, *C. sykesii* species group (Theodor and Rothschild, 1967; Graciolli and Dick, 2018). Both species consistently exhibited intraspecific morphological variation between males and females. *Cyclopodia australis* had differences in notopleural setae quantity, with 7 specimens (male and female) having one notopleural seta on each side when previous keys reported a complete lack of setae. However, COX1 sequencing of these 7 samples revealed only single-nucleotide variants in three samples, whereas the other 4 showed no notable sequence differences. Four *C. albertisii* specimens exhibited a difference in the number of barrel-like spines on male sternite 6 but showed no difference in sequencing. A small number (n=8) of *C. albertisii* females exhibited distinct dorsal abdominal setae arrangements, but no differences were observed in the COX1 sequences. Subtle interspecific colour variation was also documented between the two species, with *C. australis* appearing consistently lighter in colour for both male and female bat flies.

### Host-parasite associations and distribution patterns

*Cyclopodia albertisii* demonstrated a broad host range, parasitising all 3 flying fox hosts. This species showed a strong preference for black flying foxes and spectacled flying foxes, with 124 and 87 specimens collected, respectively. In contrast, only 3 male *C. albertisii* specimens were recovered from a single individual of the little red flying fox, suggesting limited host compatibility. *C australis* exhibited strict host specificity, being recovered exclusively from little red flying foxes (*P. scapulatus*). A total of 89 *C. australis* specimens (37 females, 52 males) were collected from this host species, representing 29.3% of the total samples collected (Table 1). Notably, no individual bat hosted multiple bat fly species simultaneously, indicating potential competitive exclusion or host-specific ecological partitioning between the 2 *Cyclopodia* species.

**Table 1.** Distribution of bat fly species based on morphological identification. Species have been separated by sex and bat host species Table 1 long description.

Count of bat flies to bat species				
Female	Black flying fox	Little red flying fox	Spectacled flying fox	Total
*C. albertisii*	59		39	99
*C. australis*		38		37
Male				
*C. albertisii*	65	3	48	116
*C. australis*		52		52
Grand Total	124	93	87	304

### Sex ratio patterns

Analysis revealed a male-to-female sex ratio of approximately 1.24:1 across both species. The male bias pattern was consistent, with *C. albertisii* showing a moderate male bias of 1.17:1 (116 males to 99 females), while *C. australis* displayed a more pronounced skew of 1.41:1 (52 males to 37 females). Overall, males comprised 55.3% of collected bat flies (168 males to 136 females), representing a departure from parity.

### Incidental observations

During specimen processing and microscopic examination, phoretic mites were incidentally collected from multiple bat fly specimens. While not quantified or systematically investigated, these mites appeared consistent with previous reports of phoretic associations between mites and nycteribiid flies (Domrow, 1961; Olival et al., 2013), suggesting complex multi-level parasitic relationships within the flying fox–bat fly system.

### Phylogenetic relationships and species validation

Maximum likelihood phylogenetic analysis of the COX1 dataset revealed 2 strongly supported monophyletic clades corresponding to the morphologically identified species *C. australis* and *C. albertisii* (Fig. 3). The *C. albertisii* clade had 100% bootstrap support, and the *C. australis* clade had 74% support, confirming species-level delineation and validating morphological identifications. The *C. australis* clade was comprised exclusively of specimens collected from little red flying foxes (*P. scapulatus*), forming a tight genetic cluster with minimal intraspecific variation. This pattern strongly supports the observed host specificity and suggests limited dispersal between host individuals or populations. The *C. albertisii* clade exhibited greater genetic conservation despite being detected from both black flying foxes (*P. alecto*) and spectacled flying foxes (*P. conspicillatus*). Although host specificity was observed for *C. australis* with little red flying foxes and *C. albertisii* with black flying foxes and spectacled flying foxes, there were no trends observed associated with collection locality and clustering of genetic relatedness.

#### Genetic variation not associated with geographical separation

Analysis within *C. albertisii* revealed no evidence of genetic variation between any individuals regardless of collection site. There was limited variation among *C. australis* individuals, though this was not attributable to collection sites across geographical regions (sites were separated by 400 km). The phylogenetic placement of outgroup sequences from related species (*C. horsfieldi* from Malaysia, *C. dubia* from Madagascar, and *C. greeffi* from Nigeria) confirmed the monophyly of both study species and supported the overall tree topology. Genetic distances between the 2 study species were consistent with species-level divergence. Kimura 2-parameter (K2P) genetic distances between *C. albertisii* and *C. australis* ranged from 1.47% to 1.66% and were consistently higher than intraspecific variation within either species (*C. albertisii*: 0%; *C.australis*: 0–0.18%), creating a clear barcoding gap of 1.29%, supporting species-level distinction (Zhang and Bu, 2022).

## Discussion

### Integrative taxonomic approach and species validation

This study represents the first comprehensive integrative assessment of bat fly diversity associated with Australian *Pteropus* species in North Queensland, combining morphological taxonomy with COX1 molecular sequencing and phylogenetic reconstruction. The identification of 2 distinct species, *C. australis* and *C. albertisii*, parasitising 3 sympatric flying fox hosts illustrates how multi-disciplinary approaches can effectively resolve ectoparasitic diversity. Maximum likelihood analysis resolved both taxa into well-supported monophyletic clades (bootstrap support >74%), with molecular identification showing strong concordance with core morphological characteristics despite subtle intraspecific variations in abdominal setae arrangement, genital structures and pigmentation patterns.

No variation was observed in COX1 among *C. albertisii* individuals, and limited variation was observed among *C. australis* individuals. The relatively close geographic proximity of sample sites, combined with the mobility of flying fox hosts, could facilitate gene flow. Within the sample range, population structure in *C. albertisii* does not appear to be influenced by geographic proximity despite this species’ broader host association and distribution. However, sampling across a larger geographic area would be necessary to determine spatial distance influences on population structure at a broader scale.

### Host specificity patterns and evolutionary implications

The contrasting host association patterns observed between the 2 *Cyclopodia* species reveal distinct evolutionary strategies. *Cyclopodia australis* exhibited a strong host association, being recovered exclusively from little red flying foxes. This pattern, reinforced by the formation of a distinct clade with only minimal intraspecific variation, suggests strong host–parasite co-adaptation and potential co-evolutionary processes. Such strict host associations are well-documented in other nycteribiid species and typically reflect long-term evolutionary relationships, niche specialisation, and strong selective pressures for host-specific adaptations (Dittmar et al., 2006; Szentiványi et al., 2019).

In contrast, *C. albertisii* demonstrated a broader host range, successfully parasitising both black flying foxes and spectacled flying foxes consistent with previous observations (Theodor and Rothschild, 1967). Black flying foxes and spectacled flying foxes are much more closely related to each other than to little red flying foxes (Almeida et al., 2020; Tsang et al., 2020), supporting the bat fly species associations that have been observed in this study. Three *C. albertisii* were collected from a single little red flying fox host that was co-housed at the Tolga Bat Hospital and had close contact with black and spectacled flying foxes at the time of sampling. Bat fly movement or misidentification of the host species could account for this unusual association.

The broader host range of *C. albertisii* is reflected in its population genetic structure, which shows no genetic differentiation between host-associated populations. The overall pattern shows 100% identical sequences from the black flying fox and spectacled flying fox hosts, indicating high levels of gene flow between host-associated populations. Previous studies have documented hidden diversity within nycteribiid and streblid bat flies, often obscured by conservative morphological evolution and pronounced sexual dimorphism (Dick and Patterson, 2006; Attaullah et al., 2023); however, our morphological data remained broadly congruent with molecular findings.

### Ecological patterns and parasite load implications

The complete absence of mixed-species infestations observed reinforces the high degree of host specificity and suggests strong reproductive isolation mechanisms or ecological barriers that prevent cross-infestation (Attaullah et al., 2023). The failure of *C. australis* to colonize black or spectacled flying foxes, despite documented roosting site overlap with little red flying foxes, further supports the existence of robust host-recognition or compatibility mechanisms.

Several host individuals carried substantial parasite loads exceeding 10 individual flies, although systematic quantification of infestation intensity was beyond the scope of this study. Such ectoparasite burdens may have significant implications for host health, stress physiology and immune function (Szentiványi et al., 2019). Given the established role of bat flies as vectors for various zoonotic pathogens, understanding parasite load dynamics is increasingly important for assessing disease transmission risks and host population health (Szentiványi et al., 2019; Xu et al., 2022).

### Sex ratio patterns and sampling considerations

The modest male bias observed in our study (1.24:1) contrasts with the female-biased patterns reported in nycteribiid bat flies (Marshall, 1981; Dick and Patterson, 2008). A comprehensive review of ectoparasitic insects found that while most species emerge with equal sex ratios, adult populations commonly exhibit female bias due to differential male mortality (Marshall, 1981). Dick and Patterson (2008) determined nycteribiid bat flies to have slightly female-biased sex ratios. This pattern may reflect several factors not systematically investigated here, such as differential sampling efficiency during handling, seasonal reproductive patterns affecting adult sex ratios, or species-specific behavioural differences in host association patterns (Dittmar et al., 2011). Temporal and spatial factors significantly influence apparent sex ratios, highlighting the importance of standardised collection protocols and suggesting rehabilitation facility sampling may not accurately reflect natural population demographics (Dittmar et al., 2011).

Several methodological constraints should be acknowledged when interpreting these results. Sampling was often limited to five specimens per host, though not consistently across carers, and quantitative infestation data were not systematically collected, as captive care conditions may have altered natural parasite loads. The relatively modest sample size for molecular analysis (50 successful sequences from 56 attempts) also limits the resolution of fine-scale population genetic patterns.

### Biogeographic significance and future directions

Despite these limitations, this study addresses a significant knowledge gap in Australian bat fly diversity and phylogenetics. Most of the global phylogenetic research on bat flies has concentrated on species from South America, Africa, and Asia (Alcantara et al., 2022; Atobatele et al., 2023; Poon et al., 2023), leaving Australian taxa severely under-sampled in comparative analyses.

The subtle morphological variations observed within these species warrant further investigation. While only one gene (COX1) was sequenced, a more robust genetic analysis, including genome-wide sequencing and additional molecular markers across broader geographic ranges, is necessary to determine whether these species represent single variable taxa or complexes of host-associated cryptic species. Such a resolution could have significant implications for uncovering hidden diversity, resolving long-standing taxonomic uncertainties, and advancing understanding of host-parasite coevolutionary dynamics within the Australasian bat fly fauna. Future studies incorporating standardised sampling protocols across seasons, host behaviours and natural roosting sites would enable stronger conclusions.

This study provides a foundation for expanded systematic surveys across Australia’s bat fauna, developing comprehensive phylogenetic frameworks, assessing conservation priorities and understanding the ecological and evolutionary processes that have shaped one of the world’s most specialised and diverse parasite–host systems.

## Conclusions

This integrative taxonomic study confirms the presence of 2 distinct *Cyclopodia* species, *C. australis* and *C. albertisii*, parasitising sympatric *Pteropus* species in North Queensland. Strong congruence between morphological identifications and COX1 phylogenetic reconstruction validates traditional taxonomic characters, despite some intraspecific variation, while demonstrating enhanced resolution through complementary approaches.

Our findings reveal contrasting evolutionary strategies: *C. australis* exhibits apparent host specificity to little red flying foxes, while *C. albertisii* demonstrates broader host compatibility across black and spectacled flying foxes. The limited genetic differentiation among host-associated *C. albertisii* populations suggests that host-mediated selection has not yet led to strong lineage diversification. However, the complete absence of mixed-species infestations indicates strong reproductive isolation mechanisms operating even among sympatric hosts, providing empirical support for ecological specialisation driving speciation in obligate parasites.

These results establish essential baseline data for disease ecology assessments. Species-specific host associations and high parasite loads (>10 flies per individual in some cases) provide critical foundations for future epidemiological studies, given the potential role of nycteribiid flies as vectors in zoonotic transmission pathways. While geographic sampling was restricted to North Queensland rehabilitation facilities, this study addresses a significant knowledge gap in Australian bat fly diversity and provides a methodological framework for expanding systematic surveys. Future priorities should include broader geographic sampling, genome-wide sequencing approaches, and comprehensive pathogen screening to fully resolve taxonomic uncertainties, assess cryptic diversity and evaluate vector competence across Australia’s understudied ectoparasite fauna.

## **Appendix I** Female Identification Key adapted and developed from Theodor and Rothschild 1967. Colours indicate different regions of the bat fly to aid in identification

**Table:** long description.

Female bat fly key	
	Bat species
A	Haltere groove completely closed by a cover.
B	Haltere groove partly covered.
C	Notopleural setae present.
D	Dorsum covered in small spines with no longer spines or setae. **No bare patch.**
E	Dorsum has 2 groups long setae at posterior lateral corners.
F	Dorsum has a group of large spines on a **bare area** in the middle.
G	Dorsum has 3−4 transverse rows of widely spaced setae with an anterior group of large spines on a bare patch.
H	Dorsum has 6 rows of long and strong setae on posterior third.
I	Dorsum has 7−12 large blunt spines & 7−12 moderately long setae posteriorly.
J	Dorsum has 9−11 long setae at posterior lateral corners and are widely spaced.
K	Dorsum has a single group of long setae 11−15 close to the central posterior border with group of spines anteriorly to long setae with no bare patch.
L	Dorsum has oblong group of spines (5−6) anteriorly and long setae (8−10) posteriorly.
M	Dorsum has oblong group of 20−30 long, thin setae in transverse rows of 4−6.
N	Dorsum has two groups of long setae (6−8) in 2 rows lateral corners to spiracle 6.
O	Dorsum posterior border has row of long setae between spiracle 6.
P	Slightly longer setae on the plurae – anterior longer than posterior above spiracle 4.
Q	Genital plate has 2 lateral sclerites separated by membranous strip which widens anteriorly and has 2 branches posteriorly.
R	Genital plate has 2 lateral sclerities – connected by a bridge to form a H.
S	Genital plate has an indistinct, narrow, median membranous stripe with short setae.
T	Genital plate is large square shape with wide membranous strip.
U	Genital plate rectangular.
V	Genital plate square.

## **Appendix II** Male identification key adapted and developed from Theodor and Rothschild 1967. Colours indicate different regions of the bat fly to aid in identification

**Table:** long description.

	Male bat fly key
	Bat species
A	Haltere groove completely closed by a cover.
B	Haltere groove partly covered.
C	Notopleural setae present.
D	Tergite 1 & 2 = 5−6 moderately **long** setae in lateral posterior corners.
E	Tergite 1 & 2 = rounded posterior margin.
F	Tergite 2 = 4−6 **short** setae at the posterior lateral corners.
G	Tergite 2 = 2 − 4 spines at the posterior lateral corners.
H	Tergite 2 = 8 short spines on posterior lateral corners.
I	Tergite 2 = 8−10 **moderately long** setae at lateral posterior margin (gap in middle).
J	Tergite 2 & 3 = only short spines in maginal rows. No long setae at all.
K	Tergite 3 = short marginal setae.
L	Tergite 3 = Straight posterior margin with moderately long setae laterally with gap in centre.
M	Tergite 4 = short marginal setae.
N	Tergite 4 = Straight posterior margin with moderately long setae laterally with gap in centre.
O	Tergite 4 = group of moderately long setae in middle with short spines on lateral margin.
P	Tergite 4 = continuous dense marginal row (possible small gap in middle).
Q	Tergite 5 = bare – no small spines.
R	Tergite 5 = Marginal row of longer setae and a narrow gap in the middle.
S	Tergite 5 = shorter than Tergite 4.
T	Tergite 5 & 6 = same length.
U	Tergite 5 = continuous marginal row of moderately long setae.
V	Tergite 5 = short marginal setae.
W	Tergite 5 = continuous dense marginal row (possible small gap in middle).
X	Tergite 6 = continuous dense marginal row (possible small gat in middle).
Y	Tergite 6 = short marginal setae.
Z	Tergite 6 = continuous marginal row of moderately long setae (may have small gap in middle).
AA	Tergite 6 = bare – no small spines.
BB	Tergite 6 = Marginal rows of longer setae and a narrow gap in the middle.
CC	Tergite 6 = shorter than Tergite 4.
DD	Tergite 6 = shorter than Tergite 5.
EE	Sternite 3 = slightly concave posterior margin with dense row of setae – longer laterally.
FF	Sternite 4 = slightly concave posterior margin with dense row of setae – longer laterally.
GG	Sternite 5 = 6 barrel shaped spines in middle.
HH	Sternite 5 = 8−10 short club-shaped spines in the middle with thin setae over rest of margin.
II	Sternite 5 = 12−15 short club-shaped spines in the middle of the hind margin.
JJ	Sternite 5 = longer than Sternite 4.
KK	Sternite 5 = moderately long setae laterally.
LL	Sternite 5 = posterior margin convex.

## Figure 1 Long description

The image A showing a brown and yellow close-up of a specimen head with dark areas, a yellow circled region on the right side and a small white label box with the letter “a” at the lower left. The image B showing an orange-brown oval structure with many small dark dots, a yellow dotted circle near the left side of the oval, several thin hair-like strands extending from the right edge and a small white label box with the letter “b” at the lower left. The image C showing an orange-brown close-up with many small dark dots, a darker central structure, multiple thin hair-like strands extending upward and outward and a small white label box with the letter “c” at the lower left. The image D showing a brown and yellow close-up of a specimen head with dark areas and a small white label box with the letter “d” at the lower left. The image E showing an orange-brown oval structure with many small dark dots, several thin hair-like strands extending from the right edge, an elongated darker structure below the oval and a small white label box with the letter “e” at the lower left. The image F showing an orange-brown close-up with many small dark dots, a darker central structure, multiple thin hair-like strands extending upward and outward and a small white label box with the letter “f” at the lower left.

Navigate back to Figure 1.

## Figure 2 Long description

The image displays two male bat fly species, C. albertisii and C. australis, with distinct morphological features. For C. albertisii male, image a shows the thorax with notopleural setae and image b shows the abdomen in dorsal view with setae present on the lateral region of tergite 3, 4, 5 and 6. For C. australis male, image a shows the thorax without notopleural setae and image b shows the abdomen in dorsal view with setae absent on tergite 3, present only on the middle region of tergite 4 and across the entire posterior edge of tergite 5 and 6.

Navigate back to Figure 2.

## Figure 3 Long description

The diagram is a maximum likelihood phylogenetic tree of Cyclopodia bat flies, oriented vertically from top to bottom. The tree scale is marked as 0.01 substitutions per site. Host species are indicated by color: blue for the little red flying fox, pink for the black flying fox and yellow for the spectacled flying fox. The tree begins with a clade supported by a bootstrap value of 74, containing sequences from Cyclopodia australis isolates associated with the little red flying fox, marked in blue. This clade includes various isolates such as D021B, G066D, D030E and others. Another clade, supported by a bootstrap value of 66, includes additional Cyclopodia australis isolates. Below, a clade supported by a bootstrap value of 80 contains Cyclopodia albertisii isolates associated with the spectacled flying fox, marked in yellow and the black flying fox, marked in pink. This clade includes isolates such as D022E, G073B, G057A and others. Further down, sequences from Cyclopodia horsfieldi, Cyclopodia greeffi and Cyclopodia dubia serve as outgroups, with bootstrap values of 100 and 98 supporting their placement. The tree visually represents the evolutionary relationships and host species associations of Cyclopodia bat flies.

Navigate back to Figure 3.

## Table 1 Long description

The table reports counts of two bat fly species by sex and by bat host species: black flying fox, little red flying fox, and spectacled flying fox, with totals. Overall, 304 bat flies were counted: 124 from black flying foxes, 93 from little red flying foxes, and 87 from spectacled flying foxes. C. albertisii dominates with 215 total, including 99 females and 116 males; it is most frequent on black flying foxes (124 combined) and also common on spectacled flying foxes (87 combined), with only 3 recorded on little red flying foxes. C. australis totals 89, with 37 females and 52 males, and is recorded only on little red flying foxes (90 combined), with none on the other two hosts. Within C. albertisii, males slightly outnumber females across hosts. One cell appears inconsistent: female C. australis on little red flying fox is listed as 38 while the row total is 37, so that value may be a transcription or counting error.

Navigate back to Table 1.

## Long description

The table is a letter-coded identification key for female bat flies, listing diagnostic anatomical features for each code from A through V. Early entries distinguish whether the haltere groove is fully closed by a cover or only partly covered, and whether notopleural setae are present. Many codes describe dorsum patterns, ranging from being covered in small spines with no bare patch to having bare areas with groups of large spines, and varying arrangements of long setae in rows or clusters. Several entries specify approximate counts and placements of long setae or blunt spines near the posterior border or posterior lateral corners. The final group of codes focuses on genital plate structure, contrasting separated lateral sclerites with a widening membranous strip, an H-shaped connection, a narrow median membranous stripe with short setae, and overall plate shapes such as rectangular or square. The table provides qualitative descriptors rather than measurements and does not map the codes to specific bat species in the data shown.

Navigate back to .

## Long description

A letter-coded identification key lists morphological characters used to distinguish male bat flies associated with bat species. Early entries describe the haltere groove as fully closed by a cover or only partly covered, and note whether notopleural setae are present. Most rows specify how many setae or spines occur on abdominal tergites one through six, including whether they are short or moderately long, whether margins are straight, rounded, concave, or convex, and whether there is a central gap. Several options contrast bare segments with segments bearing continuous dense marginal rows or marginal rows with a narrow middle gap. Additional characters describe sternites three through five, including dense rows of setae and the number and shape of spines on sternite five. The table provides character statements only and does not map letters to specific species outcomes, so it functions as a trait checklist rather than a complete decision path.

Navigate back to .
